# FCM Clustering on Interaction Pattern Analysis of Chinese Language Learner Behavior

**DOI:** 10.1155/2022/8256646

**Published:** 2022-06-08

**Authors:** Zhenzhen Yang

**Affiliations:** School of Literature, Hanjiang Normal University, Shiyan 442000, China

## Abstract

In order to meet the needs of the current personalized education, improve the shortcomings of the current digital learning system in personalized learning, and introduce the service concept into education, the development and research of the personalized learning system based on the analysis of user behavior patterns has become the development of digital learning. This paper studies a language learning method, that includes obtaining the user's language learning interaction data to determine the user's language level: wherein, the user's language level data includes the user's initial language level and the current language level; initial learning model: according to the current language level, an adaptive algorithm is used to update the initial learning model, and the user performs linguistics further according to the updated learning model. In addition, in order to realize the clustering analysis of Chinese language online learning users' learning behavior, since the Fuzzy C-means (FCM) clustering results are easily affected by the selection of their initial cluster centers, a Harmony Search (HS)-FCM-based Chinese language learning user learning behavior clustering analysis is proposed. The participation dimension, focus dimension, regularity dimension, interaction dimension, and academic performance are selected as the analysis indicators of learning behavior. The learner level is divided into 5 levels, namely excellent, good, medium, qualified, and poor. Compared with HSFCM, and decision tree, it is found that the algorithm Improved HS (HIS)-FCM in this paper has higher clustering accuracy, faster convergence speed, and lower fitness, which provides new opportunities for learner level division and optimization of course learning.

## 1. Introduction

The application of information technology to education and learning will greatly promote the development of education. As an emerging learning method in the network information age, digital learning has been gradually understood, accepted, and used by the public. With the further penetration of the Internet into every family and every classroom, various new multimedia devices have sprung up one after another. The continuous development of new educational learning software and multimedia educational and learning platforms has brought unprecedented opportunities and challenges to digital learning [1–4].

On the other hand, with the rapid development of China's economy and the continuous rise of comprehensive national strength, the depth and scale of foreign exchanges and foreign trade are deepening and expanding, and the rapid development of online teaching and online courses, the number and scale of Chinese language learning continue to increase. It has accumulated a large amount of learning data, and how to use these learning data to mine the intrinsic value of learning data to better serve the teaching and learning of Chinese language has attracted extensive attention and research. Therefore, studying the learning behavior of Chinese language learning users is of great significance for optimizing course teaching and improving course evaluation [5–9].

Personalized learning is a requirement for educational development. Constructivist learning theory, which regards learning as the process of individuals constructing their own knowledge and experience, is the mainstream of today's education. The traditional teacher-centered teaching structure is gradually becoming a new type of teaching that not only plays the leading role of teachers but also fully reflects the dominant position of students. The core is to change the status and role of teachers and students and the relationship between teachers and students. Teachers change from the masters of the classroom to the organizers and guides of classroom teaching, helping, and promoting students to construct their own knowledge. Students change from passive recipients of teaching to subjects of information processing and active constructors of knowledge. Various media have also changed from intuitive demonstration teaching aids that are only used to assist teachers in teaching to cognitive tools, collaborative communication tools, and emotional stimulation tools that can not only assist teachers in teaching but also promote students' autonomous learning and self-exploration. The characteristics of digital learning itself are based on individual active learning. Interactive curriculum design, multisensory comprehensive application of multimedia pictures, texts and sounds, and online communication and discussion create a unique environment for students to discover knowledge and cognitive construction, objectively promote the enthusiasm of students to participate in learning, and enable students to participate in learning. Using the Internet can also provide learners with a personalized learning environment, including online personal database, personal writing texts, notebooks, exercise sets, personal growth curves, and targeted teacher guidance. These thoughtful and considerate personalized learning services are accompanied by the emergence of the network, and it has promoted the rapid growth of students' knowledge ability and the healthy development of personality. Therefore, it has become a necessary research topic to design a digital learning environment that conforms to cognitive psychology and is suitable for individuals to conduct individualized learning so as to make it more conducive to learners' efficient and fast learning [10–18].

Flaws of personalized service in China's current digital learning system. Various digital learning systems emerge in an endless stream, and various educational units and education departments are spending a lot of manpower and material resources on the development of educational and teaching software, including software that can provide personalized learning. But in general, the personalization of China's personalized learning software is still only at the level of simple human-computer dialogue, and the teaching mode of human-computer dialogue is difficult to truly teach students according to their aptitude, and it is difficult to provide a relatively complete personalized education. Yes, it is difficult to adapt to the wide variety of personality differences based on simple preset question-answer patterns. What people see are mostly “standard and even” courseware of excellent teachers, lack of personality, and it is difficult to adapt to all learners of different levels and preferences, while in many online courses, the main teacher is faced with virtual reality without any immediate response. Therefore, students cannot timely adjust the teaching progress and difficulty level according to the students' responses, and can only teach according to the inherent mode. The root due to this phenomenon is that it has not changed the ancient Chinese concept of education and teaching, regarding of imparting knowledge as a gift to students, treats education itself too sacred. It does not introduce the concept of service in teaching, and it also has no awareness when it comes to education. The problem is not considered from the perspective of providing the best service for learners [19, 20].

Personalized learning system is based on user behavior pattern analysis. If education is regarded as a service to the learner, then many problems can be solved easily. Construction principle and implementation method of personalized learning system based on user behavior pattern analysis is studied. The first step in the development of a personalized learning system based on user behavior pattern analysis is to collect user data. These user data include web server access records, proxy server log records, browser log records, user profiles, registration information, user dialog or test information, user usage records, user questions, user selections, and user search keywords. After data cleaning, filtering, and data integration, through data selection, data transformation, data mining, and the use of current advanced mining technologies-Shenzhou network, rough set, machine learning, decision tree, mathematical statistics and other technologies, it can automatically discover the pattern information hidden in the data in the massive data, understand the access pattern of the system, analyze the user's behavior pattern, and automatically obtain the domain model (such as content knowledge, information processing, information resources related to user interests, and domain organization structure), user models (such as user background, interests, behaviors, and styles), and associations, and is imported into the knowledge base so as to improve the interface design for individuals, increase user traffic, and make predictive analysis. The next step is to develop a learning information recommendation service and a personalized service for personal information customization by mining the information recorded in the user's access log to grasp the user's interest and learning level. This personalized learning service platform has the ability to continuously learn from the new information collected and adapt to the dynamic changes of information and user interests so as to better provide personalized services.

In today's information society, with the rapid development of science and technology, modern, networked, informatized, and digital learning tools are widely used in teaching. E-Learning, a new learning form, is based on modern communication and communication technology to provide learners with a new learning platform. In this learning mode, learners use modern network technology to achieve interactive teaching. E-Learning is loved by learners for its autonomy, interactivity, collaboration, creativity, real-time, and other characteristics. Learners can control the pace of learning according to their own learning ability and learn through various interactive discussion methods. With the continuous development of E-Learning and the continuous update of opensource code, E-Learning's core business platform course management system and learning management system have also been greatly developed. These systems are open and shared, and can also modify and add new modules according to the user's own needs, providing learners with more free learning space.

## 2. Research on Chinese Language Learning User Learning Behavior Pattern Based on Improved Fuzzy Mean Clustering

With the accelerated iteration of the digital age, big data technology has developed rapidly in recent years. Big data opensource system software represented by distributed computing and storage systems such as Hadoop and Spark has been favored by many developers. In addition, many excellent products and technologies have been born in the generation, collection, storage, analysis, and application of data. Here, we will focus on the commonly used ELK technology stacks for data collection, organization, and retrieval: Elasticsearch, Logstash, and Kibana.

Logstash is an opensource server-side data processing pipeline tool that allows data to be extracted from multiple sources simultaneously, transformed and parsed, and then sent to any specified data repository. It is often used with NoSQL databases and highly scalable Elasticsearch.

However, Logstash has now evolved into a more general data processing pipeline tool. Logstash can process the received data as events, and the data source can be log files, e-commerce orders, customer data, chat messages, etc. These events are then processed by Logstash and sent to one or more destinations. A standard pipeline usually consists of three stages: input stage, filter stage, and output stage. Each stage can use a plugin to complete its task, and users can choose the functions of one or more stages according to their needs.

### 2.1. Input Stage

In this stage, you can understand how Logstash receives data. The input plugin can be a file plugin so that Logstash can read events from a file; it can also be used to listen to an HTTP endpoint or a relational database or even a Kafka queue. Filtering stage: the filtering stage mainly focuses on how Logstash processes the data received from the input stage. Filter plugins can parse text in CSV, XML, or JSON format. In addition, data augmentation and data enhancement work can also be carried out at this stage, such as finding and parsing its geographic location through the IP address in the data or retrieving it in relational data as an index.

### 2.2. Output Stage

The function of the output plugin is to send the processed data to the outside world, and the sending destination can be another database, file, Elasticsearch instance, or Kafka queue, etc.

Elasticsearch is an opensource distributed search and analysis engine built on Apache Lucene, and its interface is RESTful style. Since the release of Elasticsearch version 1.0 in 2010, it has quickly become the most popular full-text search engine in the industry. It is usually used in scenarios such as operational analysis, full-text search, security intelligence, and log analysis, with superior performance and continuous iteration.

The popularity of Elasticsearch is due to its powerful performance, which can support near real-time disk storage of massive data, full-text (indexed) search, and statistical analysis. Elasticsearch services are often deployed in clusters. A cluster generally contains one or more data nodes, and the data nodes contain multiple data shards, which are divided into two types: primary shards and replica shards. Each complete document will be stored in a separate data shard; the primary shard is generally preferred. The replica shard is the replication and backup of the primary shard. The two are generally deployed on different machine nodes to jointly provide external services, such as providing indexing and search functions. A complete search service generally includes the close cooperation of multiple links:

1. Parse the original document from the source field
2. Establish a mapping of documents to indexes
3. Define the data type and primary key (id) of the index
4. The text is converted into an index term through search analysis
5. Use the replica function to create replica shards to improve system high availability

Kibana is an opensource data analysis and visualization platform dedicated to application with Elasticsearch. The platform can be used for log sorting, time series data display, and application indicator warning visualization. It has built-in powerful and easy-to-use data visualization capabilities, such as heat maps, line charts, pie charts, and rich regional geographic maps on charts. In addition, it also opens an integrated interface with Elasticsearch, and its development and setup make Kibana the best choice for visualizing analysis results in Elasticsearch. The teaching process is more scientific and reasonable, and the teaching effect is optimized as a whole.

Using Kibana to create a dashboard to explore the regularity and distribution of data samples is generally divided into the following steps:

1. Add sample data and import the dataset into the Elasticsearch database
2. Define the corresponding index pattern to connect to Elasticsearch
3. Use the Discover program to match the entered query, and start searching and filtering
4. Open the Visualize program, create, and select the desired visualization type
5. Open the dashboard program, construct a suitable dashboard, and adjust the corresponding effect through the controls
6. Select the visualization area and check the actual data behind the visualization content and the corresponding query request

With the continuous progress of society and the rapid development of science and technology, the network is becoming more and more popular among the vast user groups, and there are more and more network users. Due to the individuality of each user, a large amount of behavior data will be generated in the network. What is the meaning of Internet user behavior, so far there is no unified, universally recognized, and authoritative definition. Some meaningful references are also given in some literature, and many researchers also put forward their own opinions. In a word, the network user behavior is relative to the social behavior of people in real life, and it is some activities performed by users in the virtual society of the network using computers as a tool.

In today's information society, education methods are gradually moving towards digitalization, and more and more learners tend to learn online. However, due to the abundant resources of digital learning, information may be lost. Therefore, it is necessary to provide users with information. The learning behavior is analyzed and sorted so as to extract useful information and provide it to users. User behavior analysis refers to sorting, statistic and analyzing a large amount of relevant data in the case of obtaining basic data such as website user traffic so as to discover and dig out the behavior activities of users in the process of network application. Analyze the rules and combine these rules with one or several network strategies to find out the possible problems in the current network users' learning activities and recommend resources and prompt navigation for the users to visit the website.

**Figure 1:**
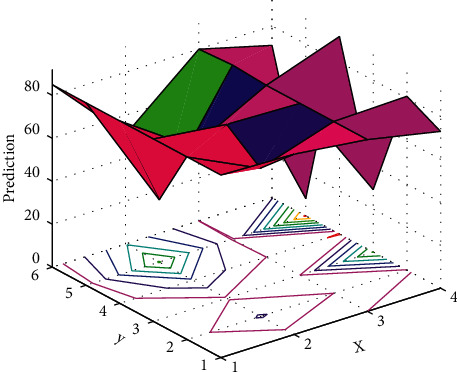
Prediction.

One of the ways to obtain user behavior is to organize and analyze the logs generated by user visits. Through data mining of the logs, we can analyze the user's learning rules so as to recommend better and more suitable resources and other information for users, and improve the efficiency of user learning. The analysis of user logs is actually a kind of knowledge discovery in the database, which is an analysis process to discover and extract some implicit, unknown, and potentially useful information from a large amount of random and fuzzy data. The process of providing these mined information to users can help users learn better and more efficiently. We can record the logs generated by user behavior into the database, analyze and organize the database, and obtain useful information for users. User behavior is divided into group user behavior and individual user behavior. Individual behavior is the behavior of a single user on the Internet, which is determined by the individual's personality, and different individual differences have different information needs. A user group composed of multiple individuals also has group user behavior. Short-term user behavior may not have obvious rules, but long-term user behavior will show a certain stability. We can use the stable regularity of user behavior to extract useful information to help users achieve higher learning efficiency. The prediction is shown in Figure 1.

Fuzzy C-means clustering is a method that uses membership to determine the category of each data sample. It has the advantages of high efficiency and small amount of calculation. However, the results of FCM clustering are easily affected by the selection of its initial clustering center. The algorithm is applied to the selection of initial clustering centers of FCM, and a clustering analysis of Chinese language learning users' learning behavior based on HIS-FCM is proposed.

If the worst and best harmony in the HS algorithm is xworst and xbest, respectively, and xworst is regarded as the base vector, the better harmony is adjusted by learning xbest. This paper proposes a method based on random position update, such as formulas (1) and (2).

(1) $$\begin{array}{c} x_{i}^{\text{new}}=x_{i}^{r}+\text{rand}\times \left(x_{d}-x_{i}^{r}\right), \end{array}$$

(2) $$\begin{array}{c} x_{d}=F\times x_{i}^{\text{best}}-x_{i}^{r}, \end{array}$$

(3) $$\begin{array}{c} x_{d}=\left\{\begin{array}{cc} x_{i}^{L}, & x^{r}<x_{i}^{L},\\ x_{i}^{U}, & x^{r}>x_{i}^{L}, \end{array}\right) \end{array}$$

where rand is a random number; xnew and *x*^*r*^ are the *i*-th dimensional variables of the newly generated harmony xnew and the randomly selected harmony *x*^*r*^, respectively; *x*^*L*^ and *x*^*U*^ are the upper and lower limits of the *i*-th dimensional variable of the harmony *x*_*i*_; *F* is the migration scale.

In order to expand the search space of the HS algorithm, reverse learning is introduced into the HS algorithm, and the reverse learning strategy is shown as follows:

(4) $$\begin{array}{c} x_{i}^{\text{new}}=\left\{\begin{array}{cc} x_{i}^{U}+x_{i}^{L}-x_{i}^{r}, & \text{rand}\leq 0.5,\\ x_{i}^{r}, & \text{other}. \end{array}\right) \end{array}$$

The small probability mutation operation in the HS algorithm is shown as follows:

(5) $$\begin{array}{c} x_{i}^{\text{new}}=x_{i}^{r}+\text{rand}\times \left(x_{i}^{U}-x_{i}^{L}\right), \end{array}$$

(6) $$\begin{array}{c} P_{m}=0.005,\text{ if rand}\leq P_{m}. \end{array}$$

The pitch fine-tuning probability PAR can be designed as

(7) $$\begin{array}{c} \text{PAR}=t\frac{{\text{PAR}}_{\max}-{\text{PAR}}_{\min}}{T}+{\text{PAR}}_{\min}, \end{array}$$

where PAR_max_ and PAR_min_ are the maximum and minimum values of the pitch fine-tuning probability.

### 2.3. Assuming Sample Data

(8) $$\begin{array}{c} x=\left\{x_{1},x_{2},\text{…},x_{n}\right\}. \end{array}$$

The sample data is *n*, and each element contains *d* attributes. The number of FCM clusters is *C* cluster center *W* = {*w*1, *w*2,…,*wc*}. Since each element category of FCM fuzzy clustering cannot be strictly divided into a specific category, let *μ* be the membership degree of the *k*-th element belonging to the *i*-th category, where:

(9) $$\begin{array}{c} \sum_{i=1}^{C}\mu_{i}=1,\mu_{i}\in \left[0,1\right]. \end{array}$$

The objective function of FCM fuzzy mean clustering is defined as

(10) $$\begin{array}{c} \min J_{m}\left(U,W\right)=\sum_{k=1}^{n}\sum_{i=1}^{C}\mu_{ik}^{2}d_{ik}^{2}. \end{array}$$

**Figure 2:**
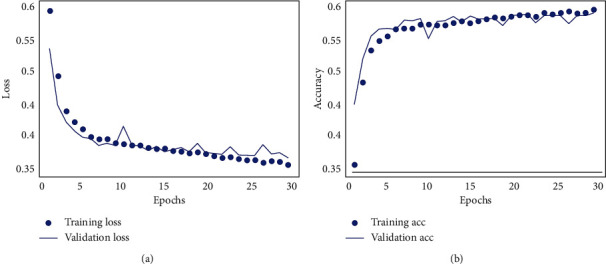
Model loss and accuracy changes (a) Loss (b) Accuracy.

In the formula, *U* is the membership degree matrix; *d*_*ik*_ represents the Euclidean distance between the element *x* and the class center *w*. The central idea of FCM fuzzy mean clustering is to continuously adjust (UW) to minimize the objective function *J* (UW). Model loss and accuracy changes are shown in Figure 2. The iterative steps of FCM fuzzy mean clustering are as follows:

**Step 1 .**

Set the number of clusters *C* and the exponential weight *b* and randomly initialize the cluster center matrix *W* so that the number of iterations *l* = 0;

**Step 2 .**

Calculate the membership degree matrix *U*;

(11) $$\begin{array}{c} u_{ik}=\left\{\begin{array}{cc} \frac{1}{\sum_{j=1}^{C}{\left(d_{ik}/d_{jk}\right)}^{2/b-1}}, & d_{ik}>0,\\ 1, & d_{ik}=0. \end{array}\right) \end{array}$$

**Step 3 .**

Correct the cluster center *W*;

(12) $$\begin{array}{c} w_{i}=\frac{\sum_{k=1}^{n}{\left(\mu_{ik}\right)}^{b}x_{k}}{\sum_{k=1}^{n}{\left(\mu_{ik}\right)}^{b}}. \end{array}$$

## 3. Empirical Analysis

User analysis is generally based on the basic attributes of the user and the specific access behavior of the user to carry out various correlation analysis or logical reasoning. There are several common methods of network user behavior analysis:

### 3.1. Statistical Analysis

Statistical analysis is the most common method of web user analysis. By analyzing the relevant data, we will obtain some statistical report information, such as the sites visited by the user, the pages browsed, the browsing time, and other information.

### 3.2. Association Analysis

Association analysis is to mine the relationship between related data in the database, that is, according to the appearance of some items in one transaction, other items will also appear in the same transaction. The example of “beer and diapers” in the famous Walmart supermarket is a typical association rule. At present, association analysis is widely used in western financial industry enterprises, and it can successfully predict the needs of bank customers, thereby improving their own marketing; other e-commerce sites also benefit from association analysis. The related algorithms of association rules include APriori algorithm, partition-based algorithm, and FP-tree frequency set algorithm. Among them, APriori algorithm is the most influential and widely used algorithm.

### 3.3. Classification Analysis

Classification is to find a conceptual description of a category, which represents the overall information of this type of data. At present, some enterprises use the method of classification analysis to improve efficiency, analyze the basic attributes and characteristics of customers, classify different customers, and predict customer purchase trends. For example, a cosmetics company will divide the cosmetics into different categories according to the customer's preference for cosmetics. When the customer buys it again, the promotion staff will distribute the brochure of the corresponding cosmetic product to the customer so as to improve the customer's interest in cosmetics. The satisfaction of the company can also greatly improve the efficiency and achieve a win-win effect for both customers and the company.

### 3.4. Cluster Analysis

Cluster analysis is to divide a set of data into several categories according to the similarity and difference. Clustering can be applied to the classification of customer groups in the enterprise, customer background analysis, customer purchase trend prediction, market segmentation, etc. In fact, clustering and classification are a reciprocal process.

### 3.5. Sequence Pattern Analysis

Sequential patterns refer to patterns with high recurrence probability searched through time series. Sequential pattern analysis is similar to association analysis, and it is also to mine the implicit relationship between data, but sequential pattern analysis focuses on the sequence relationship of data in time of occurrence, that is, causal relationship. Events occur in a certain time sequence. For example, 80% of users who viewed one resource viewed another after half an hour.

### 3.6. TOP Analysis Method

The TOP analysis method refers to the characteristics of the active user groups in the website by studying the characteristics of the most active users, such as basic attributes of users, frequently visited sites, tools, resources, etc., so as to provide users with information such as navigation assistance, recommended hot resources, etc.

User learning behavior refers to all the operation behaviors of users logging in to the digital learning platform. User behavior analysis is to analyze the user's operation behavior, dig out the implicit, unknown, and potentially useful information, and provide it to the user, giving the user a kind of guidance and help to improve the learning efficiency.

There are many user behavior analysis contents, including basic user information, website user volume, number of visits, pageviews, time of login and exit, pages viewed, time of stay, sites visited, tools accessed, and other operational behaviors. The strategies adopted in this paper for user behavior analysis are mainly divided into two directions: general usage pattern mining and personalized usage feature mining.

1. General usage pattern mining: the main target is group Web users. Through mining and analysis of the total user access behavior, frequency, content, etc., the access characteristics of group users can be obtained, which is convenient for recommending products and services to a certain characteristic group. In the study of group characteristics, this paper adopts statistical analysis and TOP analysis methods. For example, the TOP analysis method is used in a certain period of time to calculate the hot resource information in the platform; statistical analysis is used to analyze the whole learning platform at a certain time. The situation includes resources, users, etc. to make a statistic.
2. Personalized usage feature mining: the main object is every user in the network. Through mining, it analyzes each user's access behavior, frequency, content, etc., extracts the characteristics of a single user, and provides personalized e-commerce services for them. In this paper, when studying the individual characteristics of users, pattern methods such as association analysis, sequence pattern analysis, and TOP analysis are used. For example, when recommending a resource to a user, use the association analysis method to recommend other resource information accessed by other users who have visited a certain resource to the user through its relationship with other users; use sequential mode analysis to observe in a certain resource. The number of times the user has logged into the platform, which sites have been accessed, which tools have been accessed, and which resources have been accessed during a period of time is monitored. That is to find the common characteristics of a group of data objects in the database and divide them into different categories according to the classification model, which aims to attribute the data items in the database to different categories according to the classification model.

In order to verify the validity of the algorithm in this paper, the study data of the online course “Introduction to Chinese Linguistics and Philology Courses,” an open course of Netease, was selected as the research object, and the learner level was divided into 5 grades, namely excellent, good, medium, qualified, and poor, and different learning. The distribution of the type data is shown in Table 1.

In order to illustrate the effect of the cluster analysis of Chinese language online learning users' learning behavior, the evaluation indicators select the clustering accuracy rate *T* and the misjudgment rate *F*.

**Figure 3:**
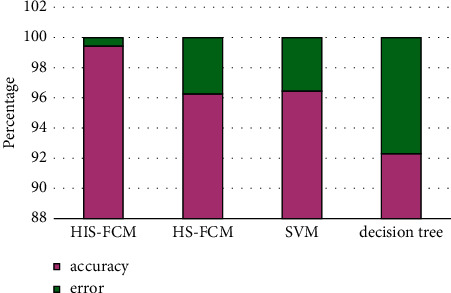
Effect comparison.

**Figure 4:**
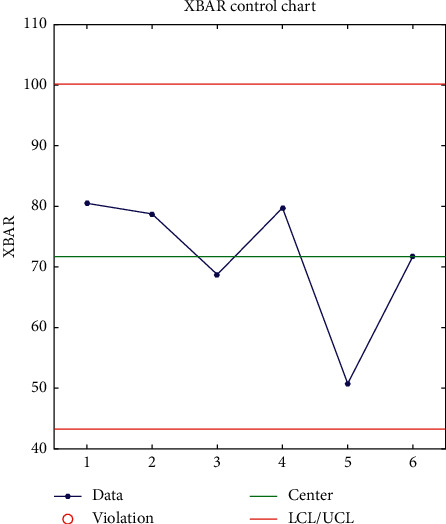
XBAR variation.

**Figure 5:**
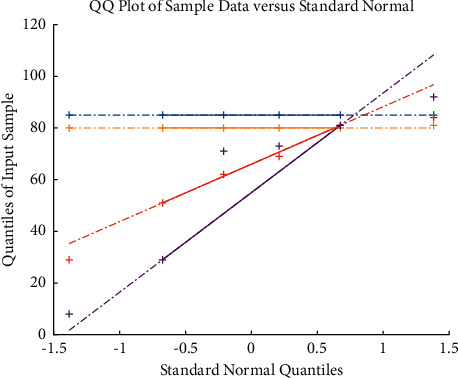
Evaluation.

**Figure 6:**
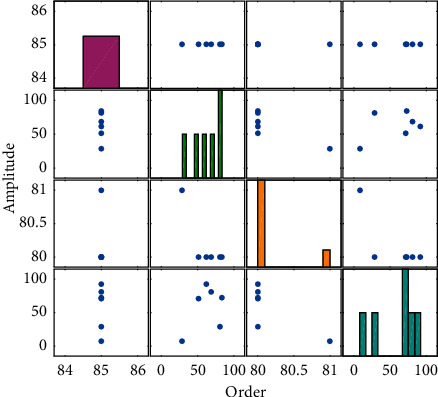
Evaluated value.

1. Accuracy rate *T*: if the number of learner types that are correctly clustered is *A*, and the actual number of learner types is *B*, then the accuracy rate of learner type clustering is the quotient of the two
2. Misjudgment rate *F*: if the actual number of learner types is *H*, and the number of misjudgments of the *i*-th learner type as the *j*-th learner type is *G*, then the number of learner types judged is *G*. The false positive rate is the quotient of the two. In order to verify the effect of the Chinese language online learning user learning behavior clustering algorithm, the algorithm IHSFCM and HSFCMSVM and the decision tree in this paper are compared as shown in Figures 3–6.

The parameters of the IHS algorithm are set as follows: the population size is 10, the maximum number of iterations is 100, and the clustering results are shown in Figure 3. The comparison of the convergence curves of IHSFCM and HSFCM is shown in Figure 6. It can be seen from Figure 6 that IHSFCM has faster convergence speed and lower fitness, and the effect is better than HSFCM.

In Figures 4–6, “^*∗*^” represents the predicted category of the learner level, and “O” represents the actual category of the learner level. Through the comparison display, the clustering results of the learner level and the actual learner level category can be visually displayed, among which 1, 2, 3, 4, and 5 indicate that the learner level is excellent, good, medium, qualified, and poor, respectively. When “^*∗*^” and “O” coincide, the predicted category at the learner level is consistent with the actual category, indicating that the clustering is correct; when “^*∗*^” and “O” do not coincide, the predicted category at the learner level is inconsistent with the actual category. At this time, the learner can cluster error. It can be seen from Figures 3–6 that the clustering accuracy and false positive rate of IHSFCM are 99.42% and 58%, respectively, which are better than 96.27% and 373% of HSFCM, 96.40% and 3.55% of SVM and 96.40% and 3.55% of decision tree. Compared with HS, FCMSVM, and decision tree, it is found that the algorithm IHSFCM in this paper has a higher clustering accuracy, which provides a new method for learner level division and optimization of course learning.

## 4. Conclusion

In order to realize the cluster analysis of Chinese language online learning users' learning behaviors, a cluster analysis of Chinese language learning users' learning behaviors based on IHSFCM is proposed in view of the fact that the FCM clustering results are easily affected by the selection of their initial cluster centers. The research results show that HIS-FCM has faster convergence speed and lower fitness than HSFCM, which provides a scientific decision-making basis for the optimization of Chinese language course learning. However, due to the lack of comprehensive consideration of the learning behavior analysis indicators in this paper, the clustering effect has the disadvantage of poor adaptability. In the future, more factors will be considered in the online learning behavior clustering analysis so as to improve the accuracy and applicability of the learning behavior analysis model.

## Figures and Tables

**Table 1:** The distribution of the type data.

Item	Training samples	Testing samples	Coding
Excellent	40	12	1
Good	40	12	2
Middle	40	12	3
Qualified	40	12	4
Poor	40	12	5

## Data Availability

The data used to support the findings of this study are available from the corresponding author upon request.
